# A second monoclinic polymorph of bis­(2,2′-bipyridine-κ^2^ *N*,*N*′)diiodido­manganese(II)

**DOI:** 10.1107/S1600536811036051

**Published:** 2011-09-14

**Authors:** Kwang Ha

**Affiliations:** a School of Applied Chemical Engineering, The Research Institute of Catalysis, Chonnam National University, Gwangju 500-757, Republic of Korea

## Abstract

The Mn^II^ ion in the title complex, [MnI_2_(C_10_H_8_N_2_)_2_], is six-coordinated in a distorted *cis*-N_4_I_2_Mn octa­hedral environment by four N atoms of the two chelating 2,2′-bipyridine ligands and two iodide anions. As a result of the different *trans* effects of the N and I atoms, the Mn—N bonds *trans* to the I atom are slightly longer than the Mn—N bonds *trans* to the N atom. The dihedral angle between the approximately planar ligands [maximum deviation = 0.064 (7) Å] is 75.0 (1)°. Numerous inter- and intra­molecular π–π inter­actions between the pyridyl rings are present, the shortest centroid–centroid distance being 3.905 (5) Å. The structure reported herein represents a new monoclinic polymorph of the previously reported monoclinic (*P*2_1_/*c*) form [Ha (2011 ▶). *Z. Kristallogr. New Cryst. Struct.* **226**, 187–188].

## Related literature

For the *P*2_1_/*c* polymorph, see: Ha (2011 ▶).
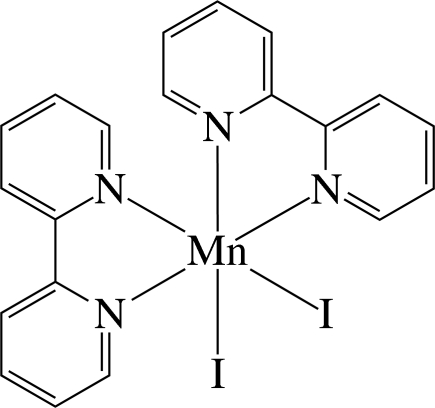


## Experimental

### 

#### Crystal data

[MnI_2_(C_10_H_8_N_2_)_2_]
*M* *_r_* = 621.11
Monoclinic, 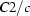

*a* = 16.491 (4) Å
*b* = 15.403 (4) Å
*c* = 17.719 (4) Å
β = 110.187 (5)°
*V* = 4224.6 (18) Å^3^
*Z* = 8
Mo *K*α radiation
μ = 3.56 mm^−1^
*T* = 200 K
0.16 × 0.13 × 0.06 mm

#### Data collection

Bruker SMART 1000 CCD diffractometer
Absorption correction: multi-scan (*SADABS*; Bruker, 2000 ▶) *T*_min_ = 0.804, *T*_max_ = 1.000
15547 measured reflections
5222 independent reflections
2330 reflections with *I* > 2σ(*I*)
*R*_int_ = 0.089

#### Refinement

*R*[*F* ^2^ > 2σ(*F* ^2^)] = 0.049
*wR*(*F* ^2^) = 0.101
*S* = 0.89
5222 reflections
244 parameters
H-atom parameters constrained
Δρ_max_ = 1.21 e Å^−3^
Δρ_min_ = −0.83 e Å^−3^

### 

Data collection: *SMART* (Bruker, 2000 ▶); cell refinement: *SAINT* (Bruker, 2000 ▶); data reduction: *SAINT*; program(s) used to solve structure: *SHELXS97* (Sheldrick, 2008 ▶); program(s) used to refine structure: *SHELXL97* (Sheldrick, 2008 ▶); molecular graphics: *ORTEP-3* (Farrugia, 1997 ▶) and *PLATON* (Spek, 2009 ▶); software used to prepare material for publication: *SHELXL97*.

## Supplementary Material

Crystal structure: contains datablock(s) I. DOI: 10.1107/S1600536811036051/ng5226sup1.cif

Structure factors: contains datablock(s) I. DOI: 10.1107/S1600536811036051/ng5226Isup2.hkl

Additional supplementary materials:  crystallographic information; 3D view; checkCIF report

## Figures and Tables

**Fig. 1.**
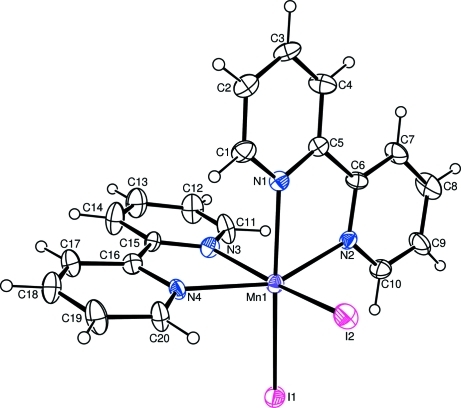
The structure of the title complex, with displacement ellipsoids drawn at the 40% probability level for non-H atoms.

**Fig. 2.**
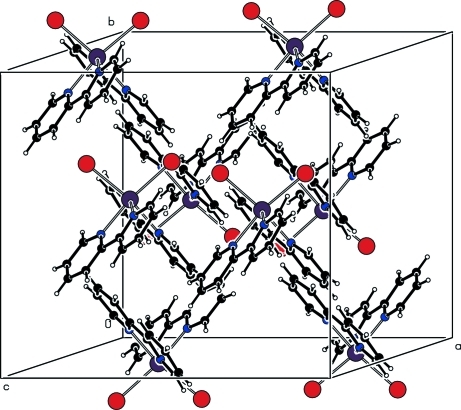
View of the unit-cell contents of the title complex.

## supplementary crystallographic information

### Comment

The crystal structure of the title complex, [MnI_2_(bipy)_2_] (bipy = 2,2'-bipyridine, C_10_H_8_N_2_), was previously reported in the monoclinic space group *P*2_1_/*c* (Ha, 2011). The structure presented herein is essentially the same as the published structure and represents a new monoclinic polymorph with the space group *C*2/*c*.

The Mn^II^ ion in the complex is six-coordinated in a considerably distorted octahedral environment by four N atoms of the two chelating bipy ligands and two iodide anions in a *cis*-N_4_I_2_ coordination geometry (Fig. 1). The tight N—Mn—N chelating angles and the I—I repelling (Table 1) contribute the distortion of the ocataheron, which results in non-linear *trans* axes [<I1—Mn1—N1 = 169.67 (14)°, <I2—Mn1—N3 = 166.94 (15)° and <N2—Mn1—N4 = 153.33 (19)°]. The Mn—I bond lengths are nearly equal, but the Mn—N bond distances occur in two distinct sets, because of the different *trans* effects of the N and I atoms (Table 1). The Mn—N bonds *trans* to the I atom are slightly longer than the Mn—N bonds *trans* to the N atom. The dihedral angle between the nearly planar bipy ligands [maximum deviation = 0.064 (7) Å] is 75.0 (1)°. The dihedral angles between the pyridyl rings containing N1 and N2 as well as N3 and N4 are 4.5 (5)° and 4.1 (5)°, respectively. In the crystal structure, the complex displays numerous inter- and intramolecular π-π interactions between the pyridyl rings, the shortest ring centroid-centroid distance being 3.905 (5) Å (Fig. 2).

### Experimental

To a solution of MnI_2_ (0.3078 g, 0.997 mmol) in EtOH (30 ml) was added 2,2'-bipyridine (0.3131 g, 2.005 mmol) and stirred for 3 h at room temperature. The precipitate was separated by filtration, washed with acetone and dried at 50 °C, to give a yellow powder (0.0854 g). Crystals suitable for X-ray analysis were obtained by slow evaporation from a methanol solution.

### Refinement

H atoms were positioned geometrically and allowed to ride on their respective parent atoms [C—H = 0.95 Å and *U*_iso_(H) = 1.2*U*_eq_(C)]. The highest peak (1.21 e Å^-3^) and the deepest hole (-0.83 e Å^-3^) in the difference Fourier map are located 1.46 Å and 0.84 Å from the atoms H14 and Mn1, respectively.

### Crystal data

**Table:**

[MnI_2_(C_10_H_8_N_2_)_2_]	*F*(000) = 2360
*M**_r_* = 621.11	*D*_x_ = 1.953 Mg m^−^^3^
Monoclinic, *C*2/*c*	Mo *K*α radiation, λ = 0.71073 Å
Hall symbol: -C 2yc	Cell parameters from 2551 reflections
*a* = 16.491 (4) Å	θ = 2.5–25.9°
*b* = 15.403 (4) Å	µ = 3.56 mm^−^^1^
*c* = 17.719 (4) Å	*T* = 200 K
β = 110.187 (5)°	Block, yellow
*V* = 4224.6 (18) Å^3^	0.16 × 0.13 × 0.06 mm
*Z* = 8	

### Data collection

**Table:**

Bruker SMART 1000 CCD diffractometer	5222 independent reflections
Radiation source: fine-focus sealed tube	2330 reflections with *I* > 2σ(*I*)
graphite	*R*_int_ = 0.089
φ and ω scans	θ_max_ = 28.3°, θ_min_ = 1.9°
Absorption correction: multi-scan (*SADABS*; Bruker, 2000)	*h* = −21→22
*T*_min_ = 0.804, *T*_max_ = 1.000	*k* = −20→14
15547 measured reflections	*l* = −23→19

### Refinement

**Table:**

Refinement on *F*^2^	Primary atom site location: structure-invariant direct methods
Least-squares matrix: full	Secondary atom site location: difference Fourier map
*R*[*F*^2^ > 2σ(*F*^2^)] = 0.049	Hydrogen site location: inferred from neighbouring sites
*wR*(*F*^2^) = 0.101	H-atom parameters constrained
*S* = 0.89	*w* = 1/[σ^2^(*F*_o_^2^) + (0.023*P*)^2^] where *P* = (*F*_o_^2^ + 2*F*_c_^2^)/3
5222 reflections	(Δ/σ)_max_ = 0.001
244 parameters	Δρ_max_ = 1.21 e Å^−^^3^
0 restraints	Δρ_min_ = −0.83 e Å^−^^3^

### Special details

**Table:**

Geometry. All e.s.d.'s (except the e.s.d. in the dihedral angle between two l.s. planes) are estimated using the full covariance matrix. The cell e.s.d.'s are taken into account individually in the estimation of e.s.d.'s in distances, angles and torsion angles; correlations between e.s.d.'s in cell parameters are only used when they are defined by crystal symmetry. An approximate (isotropic) treatment of cell e.s.d.'s is used for estimating e.s.d.'s involving l.s. planes.
Refinement. Refinement of *F*^2^ against ALL reflections. The weighted *R*-factor *wR* and goodness of fit *S* are based on *F*^2^, conventional *R*-factors *R* are based on *F*, with *F* set to zero for negative *F*^2^. The threshold expression of *F*^2^ > σ(*F*^2^) is used only for calculating *R*-factors(gt) *etc*. and is not relevant to the choice of reflections for refinement. *R*-factors based on *F*^2^ are statistically about twice as large as those based on *F*, and *R*- factors based on ALL data will be even larger.

### Fractional atomic coordinates and isotropic or equivalent isotropic displacement parameters (Å^2^)

**Table:**

	*x*	*y*	*z*	*U*_iso_*/*U*_eq_	
I1	0.12615 (3)	−0.15566 (3)	0.39194 (3)	0.03965 (16)	
I2	0.37597 (3)	−0.15176 (3)	0.36880 (3)	0.04233 (17)	
Mn1	0.24841 (7)	−0.03413 (6)	0.37953 (6)	0.0274 (3)	
N1	0.3267 (4)	0.0774 (3)	0.3538 (3)	0.0293 (14)	
N2	0.1883 (4)	0.0017 (3)	0.2492 (3)	0.0290 (16)	
N3	0.1705 (4)	0.0801 (3)	0.4074 (4)	0.0318 (15)	
N4	0.3034 (4)	−0.0032 (3)	0.5106 (3)	0.0300 (15)	
C1	0.3998 (5)	0.1097 (4)	0.4070 (5)	0.039 (2)	
H1	0.4223	0.0838	0.4587	0.047*	
C2	0.4434 (5)	0.1786 (4)	0.3898 (5)	0.040 (2)	
H2	0.4945	0.2001	0.4293	0.048*	
C3	0.4128 (5)	0.2159 (4)	0.3154 (5)	0.042 (2)	
H3	0.4420	0.2634	0.3020	0.051*	
C4	0.3378 (5)	0.1823 (5)	0.2600 (5)	0.044 (2)	
H4	0.3144	0.2075	0.2080	0.053*	
C5	0.2973 (4)	0.1128 (4)	0.2799 (4)	0.0311 (18)	
C6	0.2205 (4)	0.0702 (4)	0.2211 (4)	0.0313 (18)	
C7	0.1840 (5)	0.0976 (5)	0.1429 (4)	0.043 (2)	
H7	0.2069	0.1465	0.1246	0.052*	
C8	0.1139 (5)	0.0538 (6)	0.0911 (5)	0.057 (3)	
H8	0.0887	0.0714	0.0366	0.069*	
C9	0.0816 (7)	−0.0153 (6)	0.1198 (5)	0.060 (3)	
H9	0.0332	−0.0466	0.0855	0.072*	
C10	0.1194 (5)	−0.0384 (5)	0.1975 (5)	0.039 (2)	
H10	0.0956	−0.0861	0.2168	0.046*	
C11	0.1033 (5)	0.1199 (5)	0.3543 (4)	0.045 (2)	
H11	0.0846	0.0985	0.3008	0.054*	
C12	0.0594 (5)	0.1872 (5)	0.3691 (5)	0.052 (2)	
H12	0.0100	0.2103	0.3285	0.063*	
C13	0.0888 (6)	0.2208 (5)	0.4448 (5)	0.058 (3)	
H13	0.0607	0.2692	0.4582	0.070*	
C14	0.1609 (6)	0.1833 (5)	0.5026 (5)	0.058 (3)	
H14	0.1829	0.2061	0.5556	0.069*	
C15	0.1993 (4)	0.1125 (4)	0.4812 (4)	0.0303 (18)	
C16	0.2741 (4)	0.0669 (4)	0.5397 (4)	0.0304 (17)	
C17	0.3099 (5)	0.0904 (5)	0.6189 (4)	0.043 (2)	
H17	0.2878	0.1388	0.6388	0.052*	
C18	0.3771 (5)	0.0440 (5)	0.6689 (5)	0.047 (2)	
H18	0.4038	0.0620	0.7232	0.057*	
C19	0.4071 (5)	−0.0288 (6)	0.6417 (5)	0.052 (2)	
H19	0.4531	−0.0628	0.6762	0.063*	
C20	0.3669 (5)	−0.0496 (5)	0.5623 (4)	0.043 (2)	
H20	0.3854	−0.1004	0.5426	0.051*	

### Atomic displacement parameters (Å^2^)

**Table:**

	*U*^11^	*U*^22^	*U*^33^	*U*^12^	*U*^13^	*U*^23^
I1	0.0403 (3)	0.0408 (3)	0.0426 (3)	−0.0101 (2)	0.0204 (3)	−0.0079 (3)
I2	0.0444 (3)	0.0424 (3)	0.0452 (4)	0.0141 (3)	0.0218 (3)	0.0070 (3)
Mn1	0.0275 (6)	0.0282 (6)	0.0252 (6)	0.0012 (5)	0.0073 (5)	−0.0008 (5)
N1	0.026 (4)	0.031 (3)	0.030 (4)	0.001 (3)	0.008 (3)	0.000 (3)
N2	0.030 (4)	0.028 (4)	0.026 (4)	−0.003 (2)	0.006 (3)	−0.005 (3)
N3	0.029 (4)	0.031 (3)	0.037 (4)	0.006 (3)	0.013 (3)	−0.001 (3)
N4	0.038 (4)	0.031 (4)	0.020 (3)	0.004 (3)	0.010 (3)	0.003 (3)
C1	0.044 (5)	0.027 (4)	0.043 (5)	0.007 (4)	0.010 (4)	0.002 (4)
C2	0.033 (5)	0.033 (5)	0.049 (6)	−0.001 (4)	0.008 (4)	−0.005 (4)
C3	0.032 (5)	0.030 (4)	0.059 (6)	−0.010 (4)	0.008 (4)	−0.002 (4)
C4	0.034 (5)	0.045 (5)	0.051 (6)	−0.002 (4)	0.013 (4)	0.015 (4)
C5	0.027 (4)	0.031 (4)	0.035 (5)	0.001 (3)	0.011 (4)	0.000 (4)
C6	0.026 (4)	0.032 (4)	0.038 (5)	−0.002 (3)	0.014 (4)	0.005 (3)
C7	0.047 (5)	0.053 (5)	0.024 (5)	0.000 (4)	0.005 (4)	0.016 (4)
C8	0.040 (5)	0.091 (7)	0.023 (5)	−0.009 (5)	−0.011 (4)	0.015 (5)
C9	0.080 (8)	0.071 (6)	0.027 (5)	−0.027 (5)	0.017 (5)	0.002 (4)
C10	0.038 (5)	0.038 (5)	0.044 (5)	−0.009 (4)	0.019 (4)	−0.002 (4)
C11	0.036 (5)	0.075 (6)	0.024 (5)	0.028 (4)	0.009 (4)	0.003 (4)
C12	0.041 (5)	0.066 (6)	0.040 (6)	0.033 (4)	0.002 (4)	0.004 (4)
C13	0.056 (6)	0.065 (6)	0.054 (6)	0.021 (5)	0.019 (5)	−0.013 (5)
C14	0.060 (6)	0.067 (6)	0.037 (5)	0.027 (5)	0.007 (5)	−0.013 (4)
C15	0.029 (4)	0.033 (4)	0.025 (4)	−0.005 (3)	0.006 (4)	−0.008 (3)
C16	0.022 (4)	0.037 (4)	0.031 (5)	−0.003 (3)	0.008 (3)	−0.001 (3)
C17	0.044 (5)	0.054 (5)	0.034 (5)	0.008 (4)	0.018 (4)	−0.004 (4)
C18	0.039 (5)	0.065 (6)	0.029 (5)	0.004 (4)	0.000 (4)	−0.015 (4)
C19	0.045 (6)	0.077 (6)	0.028 (5)	0.025 (5)	0.003 (4)	0.004 (5)
C20	0.048 (5)	0.054 (5)	0.022 (5)	0.018 (4)	0.007 (4)	−0.002 (4)

### Geometric parameters (Å, °)

**Table:**

I1—Mn1	2.8149 (13)	C7—C8	1.378 (10)
I2—Mn1	2.8322 (13)	C7—H7	0.9500
Mn1—N4	2.233 (6)	C8—C9	1.365 (10)
Mn1—N2	2.245 (6)	C8—H8	0.9500
Mn1—N1	2.288 (6)	C9—C10	1.349 (10)
Mn1—N3	2.330 (5)	C9—H9	0.9500
N1—C1	1.343 (8)	C10—H10	0.9500
N1—C5	1.345 (8)	C11—C12	1.340 (10)
N2—C10	1.339 (8)	C11—H11	0.9500
N2—C6	1.350 (8)	C12—C13	1.362 (10)
N3—C15	1.325 (8)	C12—H12	0.9500
N3—C11	1.330 (8)	C13—C14	1.399 (10)
N4—C20	1.336 (8)	C13—H13	0.9500
N4—C16	1.354 (8)	C14—C15	1.378 (9)
C1—C2	1.375 (10)	C14—H14	0.9500
C1—H1	0.9500	C15—C16	1.486 (9)
C2—C3	1.364 (10)	C16—C17	1.371 (9)
C2—H2	0.9500	C17—C18	1.358 (9)
C3—C4	1.387 (9)	C17—H17	0.9500
C3—H3	0.9500	C18—C19	1.378 (10)
C4—C5	1.370 (9)	C18—H18	0.9500
C4—H4	0.9500	C19—C20	1.371 (10)
C5—C6	1.487 (9)	C19—H19	0.9500
C6—C7	1.373 (9)	C20—H20	0.9500
			
N4—Mn1—N2	153.33 (19)	C7—C6—C5	122.8 (7)
N4—Mn1—N1	89.6 (2)	C6—C7—C8	119.8 (7)
N2—Mn1—N1	71.9 (2)	C6—C7—H7	120.1
N4—Mn1—N3	71.0 (2)	C8—C7—H7	120.1
N2—Mn1—N3	87.3 (2)	C9—C8—C7	118.7 (8)
N1—Mn1—N3	82.28 (19)	C9—C8—H8	120.7
N4—Mn1—I1	96.05 (16)	C7—C8—H8	120.7
N2—Mn1—I1	99.78 (15)	C10—C9—C8	118.9 (8)
N1—Mn1—I1	169.67 (14)	C10—C9—H9	120.6
N3—Mn1—I1	91.33 (15)	C8—C9—H9	120.6
N4—Mn1—I2	99.33 (16)	N2—C10—C9	124.1 (7)
N2—Mn1—I2	99.38 (15)	N2—C10—H10	117.9
N1—Mn1—I2	89.07 (14)	C9—C10—H10	117.9
N3—Mn1—I2	166.94 (15)	N3—C11—C12	126.4 (7)
I1—Mn1—I2	98.54 (4)	N3—C11—H11	116.8
C1—N1—C5	117.9 (6)	C12—C11—H11	116.8
C1—N1—Mn1	124.8 (5)	C11—C12—C13	116.9 (7)
C5—N1—Mn1	117.4 (4)	C11—C12—H12	121.5
C10—N2—C6	117.2 (6)	C13—C12—H12	121.5
C10—N2—Mn1	124.0 (5)	C12—C13—C14	119.2 (8)
C6—N2—Mn1	118.7 (5)	C12—C13—H13	120.4
C15—N3—C11	116.8 (6)	C14—C13—H13	120.4
C15—N3—Mn1	117.2 (5)	C15—C14—C13	118.7 (8)
C11—N3—Mn1	125.7 (5)	C15—C14—H14	120.6
C20—N4—C16	117.6 (6)	C13—C14—H14	120.6
C20—N4—Mn1	122.7 (5)	N3—C15—C14	121.8 (7)
C16—N4—Mn1	119.7 (5)	N3—C15—C16	116.0 (6)
N1—C1—C2	122.8 (7)	C14—C15—C16	122.1 (7)
N1—C1—H1	118.6	N4—C16—C17	121.1 (7)
C2—C1—H1	118.6	N4—C16—C15	115.5 (6)
C3—C2—C1	119.4 (7)	C17—C16—C15	123.4 (7)
C3—C2—H2	120.3	C18—C17—C16	119.7 (7)
C1—C2—H2	120.3	C18—C17—H17	120.2
C2—C3—C4	118.0 (7)	C16—C17—H17	120.2
C2—C3—H3	121.0	C17—C18—C19	120.7 (7)
C4—C3—H3	121.0	C17—C18—H18	119.7
C5—C4—C3	120.3 (8)	C19—C18—H18	119.7
C5—C4—H4	119.9	C20—C19—C18	116.4 (7)
C3—C4—H4	119.9	C20—C19—H19	121.8
N1—C5—C4	121.5 (7)	C18—C19—H19	121.8
N1—C5—C6	115.9 (6)	N4—C20—C19	124.5 (7)
C4—C5—C6	122.5 (7)	N4—C20—H20	117.8
N2—C6—C7	121.4 (7)	C19—C20—H20	117.8
N2—C6—C5	115.8 (6)		
			
N4—Mn1—N1—C1	24.5 (6)	Mn1—N1—C5—C4	177.2 (6)
N2—Mn1—N1—C1	−175.1 (6)	C1—N1—C5—C6	175.0 (6)
N3—Mn1—N1—C1	95.3 (6)	Mn1—N1—C5—C6	−4.8 (8)
I1—Mn1—N1—C1	147.5 (7)	C3—C4—C5—N1	2.4 (12)
I2—Mn1—N1—C1	−74.9 (5)	C3—C4—C5—C6	−175.4 (7)
N4—Mn1—N1—C5	−155.7 (5)	C10—N2—C6—C7	0.1 (11)
N2—Mn1—N1—C5	4.7 (5)	Mn1—N2—C6—C7	−177.1 (6)
N3—Mn1—N1—C5	−84.9 (5)	C10—N2—C6—C5	−179.6 (6)
I1—Mn1—N1—C5	−32.7 (12)	Mn1—N2—C6—C5	3.2 (8)
I2—Mn1—N1—C5	104.9 (5)	N1—C5—C6—N2	1.1 (10)
N4—Mn1—N2—C10	−133.0 (6)	C4—C5—C6—N2	179.1 (7)
N1—Mn1—N2—C10	178.9 (6)	N1—C5—C6—C7	−178.6 (7)
N3—Mn1—N2—C10	−98.4 (6)	C4—C5—C6—C7	−0.6 (12)
I1—Mn1—N2—C10	−7.5 (6)	N2—C6—C7—C8	−1.1 (13)
I2—Mn1—N2—C10	93.0 (6)	C5—C6—C7—C8	178.6 (7)
N4—Mn1—N2—C6	43.9 (9)	C6—C7—C8—C9	1.2 (14)
N1—Mn1—N2—C6	−4.2 (5)	C7—C8—C9—C10	−0.2 (15)
N3—Mn1—N2—C6	78.6 (5)	C6—N2—C10—C9	0.9 (12)
I1—Mn1—N2—C6	169.5 (5)	Mn1—N2—C10—C9	177.9 (7)
I2—Mn1—N2—C6	−90.1 (5)	C8—C9—C10—N2	−0.9 (15)
N4—Mn1—N3—C15	6.2 (5)	C15—N3—C11—C12	−3.3 (13)
N2—Mn1—N3—C15	−158.2 (5)	Mn1—N3—C11—C12	−178.0 (7)
N1—Mn1—N3—C15	−86.0 (5)	N3—C11—C12—C13	3.3 (14)
I1—Mn1—N3—C15	102.1 (5)	C11—C12—C13—C14	−1.1 (14)
I2—Mn1—N3—C15	−37.1 (11)	C12—C13—C14—C15	−0.7 (14)
N4—Mn1—N3—C11	−179.2 (7)	C11—N3—C15—C14	1.1 (11)
N2—Mn1—N3—C11	16.5 (6)	Mn1—N3—C15—C14	176.2 (6)
N1—Mn1—N3—C11	88.6 (6)	C11—N3—C15—C16	179.7 (7)
I1—Mn1—N3—C11	−83.2 (6)	Mn1—N3—C15—C16	−5.1 (8)
I2—Mn1—N3—C11	137.6 (7)	C13—C14—C15—N3	0.8 (13)
N2—Mn1—N4—C20	−147.4 (6)	C13—C14—C15—C16	−177.7 (8)
N1—Mn1—N4—C20	−102.3 (6)	C20—N4—C16—C17	1.5 (11)
N3—Mn1—N4—C20	175.7 (6)	Mn1—N4—C16—C17	−176.3 (6)
I1—Mn1—N4—C20	86.4 (6)	C20—N4—C16—C15	−175.8 (7)
I2—Mn1—N4—C20	−13.3 (6)	Mn1—N4—C16—C15	6.4 (8)
N2—Mn1—N4—C16	30.4 (9)	N3—C15—C16—N4	−0.6 (10)
N1—Mn1—N4—C16	75.4 (5)	C14—C15—C16—N4	178.0 (7)
N3—Mn1—N4—C16	−6.6 (5)	N3—C15—C16—C17	−177.8 (7)
I1—Mn1—N4—C16	−95.9 (5)	C14—C15—C16—C17	0.8 (12)
I2—Mn1—N4—C16	164.4 (5)	N4—C16—C17—C18	1.5 (12)
C5—N1—C1—C2	2.2 (11)	C15—C16—C17—C18	178.6 (7)
Mn1—N1—C1—C2	−178.0 (5)	C16—C17—C18—C19	−3.1 (13)
N1—C1—C2—C3	−0.9 (12)	C17—C18—C19—C20	1.6 (14)
C1—C2—C3—C4	0.3 (12)	C16—N4—C20—C19	−3.2 (12)
C2—C3—C4—C5	−1.0 (12)	Mn1—N4—C20—C19	174.5 (7)
C1—N1—C5—C4	−3.0 (11)	C18—C19—C20—N4	1.7 (14)
